# Effect of single ventricular premature contractions on response to cardiac resynchronization therapy

**DOI:** 10.1186/s12872-022-02725-3

**Published:** 2022-06-25

**Authors:** Eperke Dóra Merkel, András Mihaly Boros, Walter Richárd Schwertner, Anett Behon, Attila Kovács, Bálint Károly Lakatos, László Gellér, Annamária Kosztin, Béla Merkely

**Affiliations:** Heart and Vascular Center, Semmelweis University, Varosmajor 68, Budapest, 1122 Hungary

**Keywords:** All-cause mortality, Cardiac resynchronization therapy, Premature ventricular contractions, Reverse remodeling

## Abstract

**Background:**

We lack data on the effect of single premature ventricular contractions (PVCs) on the clinical and echocardiographic response after cardiac resynchronization therapy (CRT) device implantation. We aimed to assess the predictive value of PVCs at early, 1 month-follow up on echocardiographic response and all-cause mortality.

**Methods:**

In our prospective, single-center study, 125 heart failure patients underwent CRT implantation based on the current guidelines. Echocardiographic reverse remodeling was defined as a ≥ 15% improvement in left ventricular ejection fraction (LVEF), end-systolic volume (LVESV), or left atrial volume (LAV) measured 6 months after CRT implantation. All-cause mortality was investigated by Wilcoxon analysis.

**Results:**

The median number of PVCs was 11,401 in those 67 patients who attended the 1-month follow-up. Regarding echocardiographic endpoints, patients with less PVCs develop significantly larger LAV reverse remodeling compared to those with high number of PVCs. During the mean follow-up time of 2.1 years, 26 (21%) patients died. Patients with a higher number of PVCs than our median cut-off value showed a higher risk of early all-cause mortality (HR 0.97; 95% CI 0.38–2.48; *P* = 0.04). However, when patients were followed up to 9 years, its significance diminished (HR 0.78; 95% CI 0.42–1.46; *P* = 0.15).

**Conclusions:**

In patients undergoing CRT implantation, lower number of PVCs predicted atrial remodeling and showed a trend for a better mortality outcome. Our results suggest the importance of the early assessment of PVCs in cardiac resynchronization therapy and warrant further investigations.

**Supplementary Information:**

The online version contains supplementary material available at 10.1186/s12872-022-02725-3.

## Background

Cardiac resynchronization therapy (CRT) improves cardiac function, reduces the number of hospitalizations and all-cause mortality in patients with mild to severe heart failure and a prolonged QRS [1–3]. However, the rate of non-responder patients remains relatively high [4].

The most frequent factors that can diminish effective biventricular pacing are arrhythmic events, including atrial fibrillation, premature atrial or ventricular complexes, or single beats [4]. In order to achieve the highest biventricular pacing rate and, therefore, the most beneficial response, early detection, and potential treatment of such events are essential.

In patients with a biventricular pacing rate over 98%, approximately a 44% reduction can be observed in the composite endpoint of all-cause mortality and heart failure events [5]. Although, based on prior cross-sectional analysis, only 60% of patients achieve this biventricular pacing rate [6]. One of the most frequent causes (17%) of pacing loss is premature ventricular contractions (PVCs) [6].

A subgroup analysis of the Multicenter Automatic Defibrillator Implantation Trial with Cardiac Resynchronization Therapy (MADIT-CRT) trial also showed the importance of premature ventricular and atrial complexes [7]. A relatively low frequency of ectopic beats (> 0.1%) dramatically increases the probability of low biventricular pacing (< 97%) and is associated with a higher risk of heart failure events and death along with worse echocardiographic response [7].

Accordingly, we aimed to determine the association between the early detection of single ventricular premature contractions and echocardiographic changes and all-cause mortality in patients undergoing CRT implantation.

## Methods

### Study design, patient population, and follow-up

**Fig. 1:**
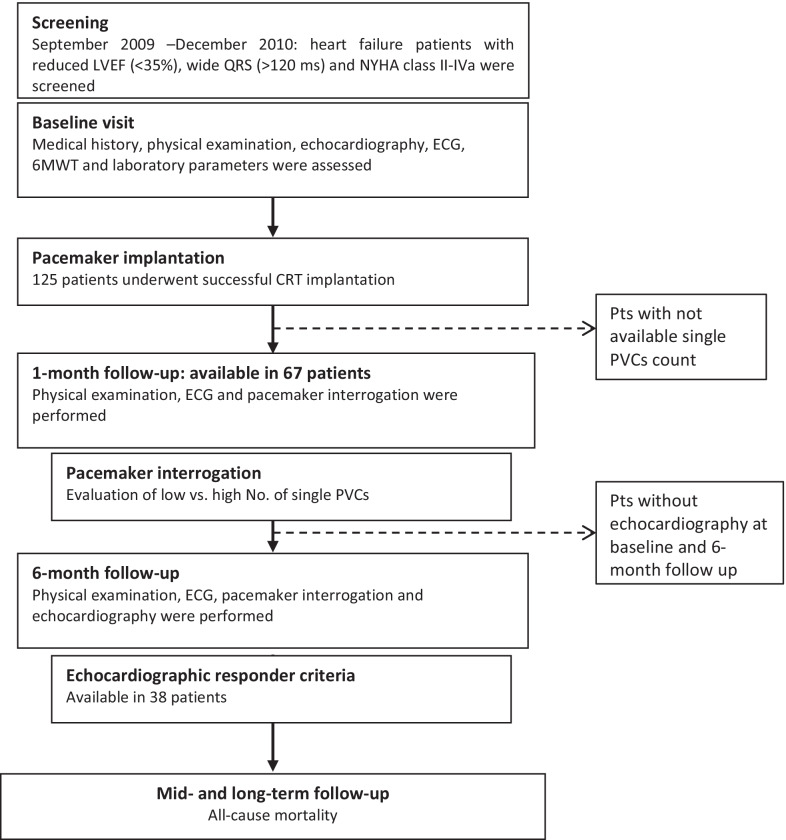
Flowchart of patient enrollment and follow-up. After successful CRT implantation in 125 patients, 1- and 6-month follow-up visits were performed, and patients were further followed for 2 years. Out of the total patient population, n = 67 patients had the complete pacemaker interrogation data and therefore were included in the final analyses. A total of thirty-eight patients had baseline and 6-month echocardiographic data and were analyzed for echocardiographic response. 6MWT: six-minute walk test; CRT: cardiac resynchronization therapy; ECG: electrocardiogram; LVEF: left ventricular ejection fraction; NYHA: New York Heart Association; PVC: premature ventricular contractions

In our prospective, observational cohort study, a total of n = 125 patients on optimal pharmacological treatment with severe chronic systolic heart failure [left ventricular ejection fraction (LVEF) ≤ 35%], wide QRS (≥ 130 ms), and ongoing symptoms [New York Heart Association (NYHA) class II-IVa] were enrolled and underwent CRT implantation (Fig. 1). Inclusion criteria met the indications of CRT of current guidelines [8], exclusion criteria included patients with known malignancies, inflammatory diseases, or genetic heart failure and those who were unable or unwilling to attend the regular follow-ups. All patients provided their written, informed consent prior to enrollment. The study was conducted in accordance with the principles of the Declaration of Helsinki and was approved by the local ethical committee. The study was performed between September 2009 and December 2010.

After successful CRT implantation, 1- and 6-months follow-up visits were performed, and patients were further followed for 4 years via phone contact. All in-person visits were scheduled 30 or 180 (± 7) days after the implantation procedures, respectively.

Detailed laboratory tests, echocardiographic examination, NYHA functional class assessment, physical examination including a six-minute walk test (6MWT) and pacemaker interrogation were performed at baseline and 6-months after CRT implantation.

All-cause mortality was assessed by the National Health Fund Death Registry index and by the regular follow-ups.

### Device implantation procedure

CRT implantation was performed according to the current guidelines. We used the subclavian transvenous approach and performed an angiogram in order to choose the ideal coronary sinus branch. Optimal lead positions were assessed by chest X-rays by using the right and left anterior oblique views. Left ventricular leads were implanted, preferably into the lateral or posterior side branch, while the right ventricular lead was recommended to be implanted in a septal position. After lead positioning, electrical parameters were measured. In patients with intraoperative phrenic nerve stimulation, repositioning was performed.

### Pacemaker interrogations and collection of ventricular premature beats

During regular follow-up visits, pacemaker interrogation was performed, and printouts were collected. The electronic database was compiled by the same physician. Ventricular, atrial and total arrhythmic events were collected from the 1- and 6-months follow-ups, and only those interrogation data were analyzed that reported the total number of single PVCs. If patients were not able to attend the 1-month follow-up visit (n = 25), the 6-month data was divided by 6.

Out of the total patient population, n = 67 patients had the complete pacemaker interrogation data and therefore were included in the final analysis (Fig. 1).

### Echocardiography

Echocardiography was performed according to current standards in a left lateral position by using the Philips iE33 echocardiography system equipped with an S5-1 transducer (Philips Healthcare, Best, The Netherlands). Image acquisition was performed according to the current recommendations [9]. Measurements were performed offline by using the QLAB software (Philips Healthcare). Left ventricular end-systolic and end-diastolic volumes (LVESV and LVEDV) were measured, and LVEF was calculated by the biplane Simpson’s method. Left atrial volume (LAV) was measured by monoplane Simpson’s method from apical four-chamber or two-chamber view in end-systole, whichever was available [9].

### Endpoints

The primary endpoint was all-cause mortality during the follow-up period, which was evaluated in n = 67 patients.

Secondary endpoints included three echocardiographic response criteria defined as at least a 15% relative improvement in LVEF, or at least a 15% decrease in LVESV, or at least a 15% decrease in LAV 6 months after CRT implantation. A total of thirty-eight patients had baseline and 6-month echocardiographic data and were analyzed for echocardiographic response (Fig. 1).

### Statistical analysis

A two-sided *P*-value of < 0.05 was considered statistically significant in all cases. Statistical analyses were carried out by using the IBM SPSS version 22 software (IBM Corp. Released 2013. IBM SPSS Statistics for Windows, Version 22.0. Armonk, NY: IBM Corp) and Graphpad Prism 6.03 (Graph-Pad Softwares Inc., USA) software.

The normality of the data was checked via the Shapiro–Wilk test. Continuous variables were presented as mean ± standard deviation (SD), or as median with interquartile range (IQR, 25–75%), as appropriate. Categorical data were described with frequency and percentage. Baseline clinical characteristics were compared by unpaired t-test, or Mann–Whitney U-test, as relevant. The Fisher’s exact test was used for comparison of categorical data.

For the later analyses, we chose a cut-off point (the median value of PVCs and 15,000 beats shown in Additional file 1) that had appropriate sensitivity, specificity, and clinical relevance. Based on the median cut-off point, the patients were divided into “low” and “high PVCs” groups. Time-to-event data were analyzed by the Gehan-Breslow-Wilcoxon test, since this method gives more weight to deaths at early time points as compared to the log-rank test.

Univariate Cox was performed to reveal the predictors of mortality. Adjusted hazard (HR) with 95% confidence intervals (CI) were calculated for all-cause mortality via logistic regression analyses as a forward stepwise way.

## Results

### Baseline clinical characteristics

The mean age of the patients (n = 67) was 66.2 ± 10.2 years, 52% had ischemic etiology of heart failure, and the mean LVEF was 29.0 ± 6.0%. The electrocardiogram (ECG) showed typical left bundle branch block (LBBB) morphology in 73% of the cases (Table 1).

**Table 1:** Baseline clinical variables, medical history, echocardiographic measurements, medical therapy and laboratory parameters

Baseline clinical variables	All patients (n = 67)	Low PVCs (n = 34)	High PVCs (n = 33)	*P*-value
No. of single PVCs (no., IQR)	11,401 (725/48 K)			
Age (years, mean ± SD)	66.2 ± 10.2	64.5 ± 11.3	68.5 ± 8.4	0.16
Gender (female, n, %)	14 (21%)	10 (29%)	4 (12%)	0.13
Ischemic etiology (n, %)	35 (52%)	16 (47%)	19 (58%)	0.47
NYHA (stadium, mean ± SD)	3.2 ± 2.0	3.1 ± 2.0	3.3 ± 2.0	0.21
QRS (ms, mean ± SD)	162 ± 24	168 ± 25	157 ± 22	0.10
typical LBBB morphology (n, %)	49 (73%)	26 (77%)	23 (70%)	0.59
not typical LBBB (n, %)	18 (27%)	8 (24%)	10 (30%)	0.59
6MWT (m, mean ± SD)	295.9 ± 125.7	318.0 ± 119.6	276.3 ± 129.9	0.23
RR systolic (mmHg, mean ± SD)	121.9 ± 18.3	121.4 ± 18.3	122.5 ± 18.1	0.81
RR diastolic (mmHg, mean ± SD)	74.1 ± 10.2	73.3 ± 9.6	74.9 ± 10.9	0.52
Heart rate (min^−1^, mean ± SD)	73.4 ± 13.4	72.3 ± 12.0	74.6 ± 14.8	0.52
Sinus rhythm (n, %)	55 (82%)	29 (86%)	26 (79%)	0.54
*Medical history*				
Hypertension (n, %)	46 (69%)	23 (68%)	23 (70%)	1.00
Type 2 diabetes mellitus (n, %)	22 (33%)	12 (35%)	10 (30%)	0.78
Prior myocardial infarction (n, %)	17 (25%)	10 (29%)	7 (21%)	0.58
Prior PCI (n, %)	17 (25%)	9 (27%)	8 (24%)	1.00
Prior CABG (n, %)	10 (15%)	3 (9%)	7 (21%)	0.19
Prior COPD (n, %)	4 (6%)	1 (3%)	3 (9%)	0.36
*Echocardiographic parameters*				
LVEF (%, mean ± SD)	29.0 ± 6.0	30.4 ± 6.7	27.7 ± 5.1	0.14
LVESV (ml, mean ± SD)	183.8 ± 68.1	170.7 ± 63.0	196.8 ± 72.1	0.24
LAV (ml, mean ± SD)	87.6 ± 26.8	94.1 ± 25.6	81.9 ± 27.3	0.18
*Baseline medical therapy*				
Beta blocker (n, %)	61 (91%)	32 (94%)	29 (88%)	0.43
ACE inhibitor or ARB (n, %)	63 (94%)	32 (94%)	31 (94%)	1.00
MRA (n, %)	44 (66%)	22 (65%)	22 (67%)	1.00
Diuretics (n, %)	55 (82%)	24 (88%)	31 (94%)	0.06
Digoxin (n, %)	15 (22%)	8 (24%)	7 (21%)	1.00
Amiodarone (n, %)	17 (25%)	12 (35%)	5 (15%)	0.09
Oral anticoagulant therapy (n, %)	21 (31%)	8 (24%)	13 (39%)	0.19
*Baseline laboratory parameters*				
Sodium (mmol/L, mean ± SD)	138.6 ± 2.7	139.0 ± 2.5	138.1 ± 2.8	0.18
Potassium (mmol/L, mean ± SD)	4.6 ± 0.5	4.6 ± 0.6	4.5 ± 0.4	0.33
Creatinine (μmol/L, mean ± SD)	110.1 ± 44.1	120.7 ± 52.2	99.2 ± 30.9	0.07
BUN (mmol/L, mean ± SD)	9.8 ± 5.3	9.7 ± 4.0	9.8 ± 6.4	0.23

6MWT, 6-min walk test; ACE, angiotensin converting enzyme inhibitor; ARB, angiotensin receptor blocker; BUN, blood urea nitrogen; CABG, coronary artery bypass graft; COPD, chronic obstructive pulmonary disease; IQR, interquartile range; LAV, left atrial volume; LBBB, left bundle branch block; LVEDV, left ventricular end-diastolic volume; LVEF, left ventricular ejection fraction; LVESV, left ventricular end-systolic volume; MRA, mineralocorticoid receptor antagonist; NYHA, New York Heart Association; PCI, percutaneous coronary intervention; PVCs, premature ventricular contractions; RR, Riva Rocci; SD, standard deviation

The median value of single PVCs at the 1-month follow-up visit was 11,401 in our patient population. Patients with a lower number of PVCs than 11,401 were categorized as “low PVCs”, while patients with a higher number of PVCs at the 1-month follow-up visit were categorized as “high PVCs”.

There were no statistically significant differences between the two groups as regards to baseline clinical parameters, medical history or echocardiographic parameters (Table 1). There were no relevant differences in terms of baseline medication, similar pharmacological regime was used in the two groups (Tables 1 and 2). Also renal function parameters as serum creatinine (120.7 ± 52.2 μmol/L vs. 99.2 ± 30.9 μmol/L, *P* = 0.07) and blood urea nitrogen levels were similar in the two groups (9.7 ± 4.0 mmol/L vs. 9.8 ± 6.4 mmol/L, *P* = 0.23). Serum potassium levels that might influence arrhythmic events or the number of premature beats were also similar in the two groups (4.6 ± 0.6 vs. 4.5 ± 0.4 mmol/L, *P* = 0.33).

**Table 2:** Type and dose of baseline beta blockers

Baseline beta blocker therapy	Low PVCs (n = 34)	High PVCs (n = 33)	*P* value
Carvedilol (n, %)	8 (25%)	8 (28%)	1.00
Mean dose of cardvedilol (mg, mean ± SD)	21.3 ± 8.8	19.5 ± 14.0	0.55
Bisoprolol (n, %)	11 (69%)	13 (45%)	0.44
Mean dose of bisoprolol (mg, mean ± SD)	4.3 ± 3.0	4.1 ± 1.7	0.65
Metoprolol (n, %)	9 (27%)	6 (18%)	0.56
Mean dose of metoprolol (mg, mean ± SD)	40.3 ± 26.4	54.2 ± 24.6	0.23
Nebivolol (n, %)	4 (12%)	1 (3%)	0.36
Mean dose of nebivolol (mg, mean ± SD)	5.0 ± 0	5.0 ± 0	1.00

The median value of single PVCs at 1-month follow-up visit was 11,401 in our patient population. Patients with a lower number of PVCs than 11,401 were categorized as “low PVCs”, while patients showing more than 11,401 PVCs at 1-month follow-up visit were categorized as “high PVCs”

PVCs, premature ventricular contractions; SD, standard deviation

Biventricular pacing rate did not differ significantly in patients with “low PVCs” and “high PVCs” at the 1-month follow up [100% (99 / 100%) vs. 99.5% (94.5 / 100%),

*P* = 0.13] indicating that the amount of PVCs in this range did not influence the biventricular pacing rate.

### Prognosis and clinical outcome by the amount of PVCs at 1-month follow-up

**Fig. 2:**
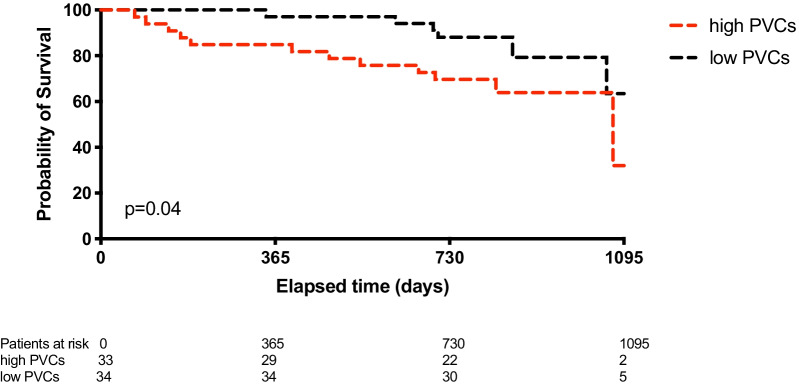
Survival of patients with low versus high PVCs. The median value of single PVCs at 1-month follow-up visit was 11,401 in our patient population. Patients with a lower number of PVCs than 11,401 were categorized as “low PVCs”, while patients showing more than 11,401 PVCs at 1-month follow-up visit were categorized as “high PVCs”. In the “low PVCs” group, n = 7 patients passed away, while in the “high PVCs” group, 12 patients reached the primary endpoint (*P* = 0.04). PVC: premature ventricular contractions

During the mean follow-up time of 2.1 years, 19 (28%) patients died. In the “low PVCs” group n = 7 patients passed away, while in the “high PVCs” group n = 12 patients reached the primary endpoint (HR 0.97; 95% CI 0.38–2.48; *P* = 0.04) (Fig. 2). While during the long-term follow-up, a mean of 6.8 years, 40 (60%) patients died, 19 versus 21 reached the primary endpoint, respectively, which showed no significant difference between the two groups (HR 0.78; 95% CI 0.42–1.46; *P* = 0.15).

### Association of the prevalance of PVCs at 1-month follow up and 6-month echocardiographic changes

We analyzed echocardiographic changes 6 months after CRT implantation in thirty-eight patients in “low” versus “high PVCs” groups. Left ventricular parameters were similar in the 2 groups (LVEF + 9.1 ± 6.6 vs. + 8.6 ± 8.7; *p* = 0.89) (LVESV − 39.0 ± 50.4 vs. − 46.4 ± 50.2; *p* = 0.82).

**Fig. 3:**
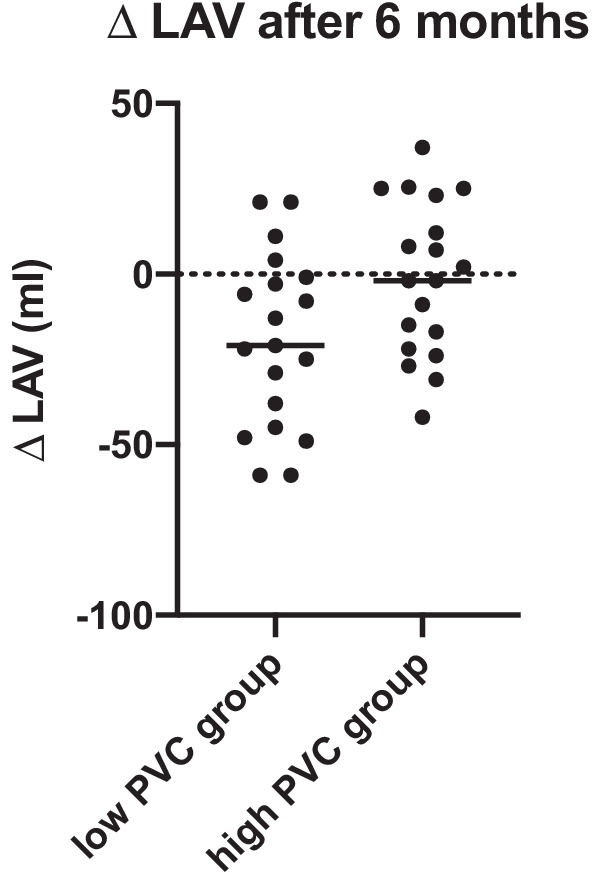
Difference in LAV changes after 6 months in patients with low versus high PVCs

At the same time, the decrease of LAV was significantly higher in the “low PVCs” group compared to the “high PVCs” group (− 19.4 ± 25.4 vs. − 1.4 ± 22.5; *p* = 0.02) (Table 3 and Fig. 3).

**Table 3:** Changes of echocardiographic parameters 6 months after CRT implantation

Echocardiographic changes	All patients (n = 38)	Low PVCs (n = 19)	High PVCs (n = 19)	*P*-value
Δ LVEF (%, mean ± SD)	+ 8.8 ± 7.6	+ 9.1 ± 6.6	+ 8.6 ± 8.7	0.89
Δ LVESV (mL,mean ± SD)	− 42.7 ± 49.7	− 39.0 ± 50.4	− 46.4 ± 50.2	0.82
Δ LAV (mL, mean ± SD)	− 10.4 ± 25.4	− 19.4 ± 25.4	− 1.4 ± 22.5	**0.02**

Bold indicate statistically significant as in the p-value is < 0.05

The median value of single PVCs at one-month follow-up visit was 11,401 in our patient population. Patients with a lower number of PVCs than 11,401 were categorized as “low PVCs”, while patients showing more than 11,401 PVCs at the one-month follow-up visit were categorized as “high PVCs”

A total of thirty-eight patients had baseline and six-month echocardiographic data and were analyzed for echocardiographic changes (Δ)

CRT, cardiac resynchronization therapy; LAV, left atrial volume; LVEF, left ventricular ejection fraction; LVESV, left ventricular end-systolic volume; PVCs, premature ventricular contractions; SD, standard deviation

## Discussion

We found that the number of PVCs 1 month after CRT implantation has impact on the echocardiographic left atrial reverse remodeling, while has moderate—if any—influence on mortality in our patient cohort.

CRT is an effective therapy in symptomatic patients with chronic systolic heart failure, low ejection fraction, and wide QRS, but the beneficial response is multifactorial; it strongly depends on optimal patient selection, electrical parameters at implantation, and the biventricular pacing rate [1–3]. Based on a large cohort study of CRT candidates by Cheng et al., only 40% of patients have more than 98% biventricular pacing rate [6]. Between 95 and 98% pacing rate, the most frequent cause that results in loss of optimal pacing is the elevated number of single ventricular premature contractions, which affects 18.7% of the patients [6]. In our study, the effectiveness of biventricular pacing was not diminished by PVCs, but we observed a less favorable outcome, atrial reverse remodeling in patients with a high number of PVCs.

Ruwald et al. also found similar results: patients with a relatively low burden of ectopic beats (as low as 1 in 1.000) are more likely to have a worse echocardiographic response and clinical outcome (incidence of ventricular tachyarrhythmias and all-cause death). In the above-mentioned MADIT-CRT substudy, ectopic beats (atrial and ventricular premature complexes together) with an occurrence as low was 0.1% were linked to poorer reverse remodeling and increased heart failure events [7]. In our study PVCs solely made up 0.4% of heartbeats (in case of the median of 11,401). We did not find a statistically significant correlation between the 6-month changes of left ventricular dimensions and the 1-month number of PVCs, but the number of PVCs predicted the atrial reverse remodeling by atrial voume measurements. This beneficial effect of low prevalence of PVCs was also described in 47 patients after PVC ablation by Akkaya et al. [10]. Six months after PVC ablation, a higher left atrial volume reduction and improvement of diastolic function were seen independently of LVEF in patients with a lower baseline number of PVCs [10]. Moreover, Park et al. also found that LAV index correlates well with PVC burden and PVC burden predicts LAV index independently of age, sex, and comorbidities [11].

While further studies also raised the question whether the origin of PVCs might have an impact and influence on the subsequent echocardiographic reponse. Wojdyła-Hordyńska et al. described after investigating 110 consecutive patients underwent monomorphic PVC ablation from an outflow tract origin or from the left ventricle, only outflow tract PVC elimination predicted left ventricular improvement in 6 months [12].

The effect of CRT on left ventricular and atrial reverse remodeling has been studied previously. Left atrial reverse remodeling may be due to the synchronous contraction leading to a better left ventricular filling, an increased cardiac output and decreased mitral regurgitation [13, 14]. Both LAV and LVESV reductions independently decrease the risk of HF and death [15, 16]. Some patients experience only atrial reverse remodeling and they have comparable outcome with complete left sided reverse remodeling (HR 2.0; 95% CI 0.7–5.6; *P* = 0.21) These patients have intermediate outcomes in both echocardiographic, and long-term mortality and HF hospitalizations, supposedly due to an improved left ventricular diastolic filling [17].

In a MADIT-CRT subanalysis by Mathias et al., 22% of the patients underwent CRT implantation had discordant left sided reverse remodeling (either LAV or LVESV reduction). Those with complete left sided reverse remodeling had a significantly lower rate of HF and death compared to the discordant and lesser reverse remodeling patient group. But, the discordantly reverse remodeled patients had better outcomes compared to those in the lesser reverse remodeling group. Predictive factors of complete reverse remodeling were sex (female), non-ischemic etiology, and a lower percent of unfavorbale clinical parameters (lower LAV and LVESV, higher LVEF) [18]. In our study we did not experience significant differences in these clinical baseline characteristics.

Considering the above-mentioned results, we do not know whether, in such a relatively low prevalence of PVCs, is it a cause or symptom of e.g. a more activated sympathetic nervous system? Could it be relevant to decrease the number of PVCs and if so, what would be the proper method (ablation or drugs)?

While PVCs can facilitate heart failure progression in patients with CRT due to the loss of effective biventricular pacing, their hemodynamic effect can also be relevant considering the impaired systolic and diastolic function. Even though the cause and consequences of PVCs, and most relevantly their clinical implications are ambiguous, but the early identification of patients with a higher number of PVCs, as well as their close follow-up and maximized medical treatment might be beneficial.

## Conclusions

The early assessment of single premature ventricular contractions 1 month after CRT implantation shows association with 6-month left atrial reverse remodeling and presumably of a better outcome.

## Limitations

Our study is limited by the relatively small sample size and the low number of endpoints. Nonetheless, our results are in line with other large, multicenter, randomized trials. Second of all, when pacemaker interrogation files of the 1-month follow-up were not available (n = 25), the 6-month data was divided by six. The implanted CRT devices varied in brand and type, thus present a limitation due to their different PVC detection modes. Moreover it should be noted that PVC burden may be altered by atrial fibrillation episodes, fused beats, and supraventricular contractions with aberration. This analysis is mainly hypothesis-generating, and the results should be regarded, therefore as preliminary. More extensive studies are needed to confirm the results and the clinical impact of PVCs in patients undergoing CRT implantation.

## Supplementary Information

**Additional file 1: Figure S1**. PVC distribution of enrolled patients at 1-month, 6-month and total PVC number. **Figure S2**. Difference in LAV changes after 6 months in patients with low vs. high PVCs, with 15000 beats as cut-off value. (low PVC group (mean ± SD): − 18.4 ± 26.6 ml vs. high PVC group (mean ± SD): − 0.56 ± 20.6 ml; p = 0.029). **Table 1**. Baseline clinical variables, medical history, echocardiographic measurements, medical therapy and laboratory parameters of patients dichotomized by 15000 beats as cut-off value value

## Data Availability

The data that support the findings of this study are available from Annamária Kosztin, MD, PhD (kosztin.annamaria@med.semmelweis-univ.hu) but restrictions apply to the availability of these data, which were used under license for the current study, and so are not publicly available. Data are however available from the authors upon reasonable request and with permission of Semmelweis University.

## Abbreviations

ACE: Angiotensin converting enzyme inhibitor
ARB: Angiotensin receptor blocker
BUN: Blood urea nitrogen
CABG: Coronary artery bypass graft
COPD: Chronic obstructive pulmonary disease
CI: Confidence interval
CRT: Cardiac resynchronization therapy
HR: Hazard ratio
IQR: Interquartile range
LAV: Left atrial volume
LBBB: Left bundle branch block
LVEF: Left ventricular ejection fraction
LVEDV: Left ventricular end-diastolic volume
LVESV: Left ventricular end-systolic volume
MRA: Mineralocorticoid receptor antagonist
NYHA: New York Heart Association
OR: Odds ratio
PCI: Percutaneous coronary intervention
PVC: Premature ventricular complex
ROC: Receiver-operator characteristic
RR: Riva Rocci
SD: Standard deviation
6-MWT: 6-Minute walk test
