# Supplementary material for: Effect of single ventricular premature contractions on response to cardiac resynchronization therapy
Source: BMC Cardiovasc Disord. 2022 Jun 25;22:289. doi: 10.1186/s12872-022-02725-3 (PMC9233778; doi:10.1186/s12872-022-02725-3)
Supplement: Supplementary file 1 — Additional file 1: Figure S1. PVC distribution of enrolled patients at 1-month, 6-month and total PVC number. Figure S2. Difference in LAV changes after 6 months in patients with low vs. high PVCs, with 15000 beats as cut-off value. (low PVC group (mean ± SD): − 18.4 ± 26.6 ml vs. high PVC group (mean ± SD): − 0.56 ± 20.6 ml; p = 0.029). Table 1. Baseline clinical variables, medical history, echocardiographic measurements, medical therapy and laboratory parameters of patients dichotomized by 15000 beats as cut-off value value [file 12872_2022_2725_MOESM1_ESM.docx]

**Supplementary material**

**Supplementary Figure 1.** PVC distribution of enrolled patients at 1-month, 6-month and total PVC number

**Supplementary Figure 2.** Difference in LAV changes after 6 months in patients with low vs. high PVCs, with 15000 beats as cut-off value. (low PVC group (mean±SD): -18.4 ± 26.6 ml

vs. high PVC group (mean±SD): -0.56 ± 20,6 ml; p=0.029)

| **Table 1Supplementary table 1.** Baseline clinical variables, medical history, echocardiographic measurements, medical therapy and laboratory parameters of patients dichotomized by 15000 beats as cut-off value value | | | | |
| --- | --- | --- | --- | --- |
| **Baseline clinical variables** | **All patients**  **(n = 67)** | **low PVCs (n = 38)** | **high PVCs (n = 29)** | ***P*-value** |
| Age (years, mean ± SD) | 66.2 ± 10.2 | 64.5 ± 10.7 | 68.5 ± 9.1 | 0.11 |
| Gender (female, n, %) | 14 (21%) | 11 (29%) | 3 (10%) | 0.07 |
| Ischemic etiology (n, %) | 35 (52%) | 20 (53%) | 15 (52%) | 0.99 |
| NYHA (stadium, mean ± SD) | 3.2 ± 2.0 | 3.1 ± 1.9 | 3.3 ± 2.1 | 0.63 |
| QRS (ms, mean ± SD) | 162 ± 24 | 164 ± 26 | 159± 23 | 0.45 |
| typical LBBB morphology (n, %) | 49 (73%) | 28 (74%) | 21 (72%) | 0.99 |
| 6MWT (m, mean ± SD) | 295.9 ±125.7 | 322.0 ± 119.1 | 270.7 ± 129.0 | 0.14 |
| RR systolic (mmHg, mean ± SD) | 121.9 ± 18.3 | 121.5 ± 18.2 | 122.4 ± 18.2 | 0.85 |
| RR diastolic (mmHg, mean ± SD) | 74.1 ± 10.2 | 72.2 ± 10.0 | 76.4± 10.2 | 0.09 |
| Heart rate (min^-1,^ mean ± SD) | 73.4± 13.4 | 71.3± 11.8 | 76.1 ± 14.9 | 0.18 |
| Sinus rhythm (n, %) | 55 (82%) | 33 (87%) | 22 (76%) | 0.34 |
| **Medical history** |  |  |  |  |
| Hypertension (n, %) | 46 (69%) | 26 (68%) | 20 (69%) | 0.99 |
| Type 2 diabetes mellitus (n, %) | 22 (33%) | 13 (34%) | 9 (31%) | 0.99 |
| Prior myocardial infarction (n, %) | 17 (25%) | 12 (31%) | 5 (17%) | 0.26 |
| Prior PCI (n, %) | 17 (25%) | 10 (26%) | 7 (24%) | 0.99 |
| Prior CABG (n, %) | 10 (15%) | 5 (13%) | 5 (17%) | 0.73 |
| Prior COPD (n, %) | 4 (6%) | 2 (5%) | 2 (7%) | 0.99 |
| **Echocardiographic parameters** |  |  |  |  |
| LVEF  (%, mean ± SD) | 29.0 ±6.0 | 29.9 ± 6.9 | 27.9 ± 4.7 | 0.31 |
| LVESV  (ml, mean ± SD) | 183.8 ± 68.1 | 176.6 ± 75.7 | 192.6 ± 58.4 | 0.49 |
| LAV (ml, mean ± SD) | 83.8 ± 25.2 | 79.6 ± 24.6 | 88.9 ± 25.8 | 0.26 |
| **Baseline medical therapy** |  |  |  |  |
| Beta blocker (n, %) | 61 (91%) | 35 (92%) | 26 (89%) | 0.99 |
| ACE inhibitor or ARB (n, %) | 63 (94%) | 35 (92%) | 28 (96%) | 0.63 |
| MRA (n, %) | 44 (66%) | 24 (63%) | 20 (69%) | 0.79 |
| Diuretics (n, %) | 55 (82%) | 27 (71%) | 28 (96%) | **0.01** |
| Digoxin (n, %) | 15 (22%) | 9 (24%) | 6 (21%) | 0.99 |
| Amiodarone (n, %) | 17 (25%) | 13 (34%) | 4 (14%) | 0.08 |
| Oral anticoagulant therapy (n, %) | 21 (31%) | 10 (26%) | 11 (38%) | 0.43 |
| **Baseline laboratory parameters** |  |  |  |  |
| Sodium (mmol/L, mean ± SD) | 138.6 ± 2.7 | 139.0 ± 2.5 | 138.1 ± 2.8 | 0.15 |
| Potassium (mmol/L, mean ± SD) | 4.6 ± 0.5 | 4.5 ± 0.6 | 4.6 ± 0.3 | 0.69 |
| Creatinine (μmol/L, mean ± SD) | 110.1 ± 44.1 | 119.7 ± 52.2 | 97.4 ± 26.0 | **0.04** |
| BUN (mmol/L, mean ± SD) | 9.8 ± 5.3 | 10.14 ± 5.7 | 9.2 ± 4.7 | 0.48 |
| 6MWT: 6-minute walk test; ACE = angiotensin converting enzyme inhibitor; ARB = angiotensin receptor blocker; BUN = blood urea nitrogen; CABG = coronary artery bypass graft; COPD = chronic obstructive pulmonary disease; IQR = interquartile range; LAV: left atrial volume; LBBB = left bundle branch block; LVEDV: left ventricular end-diastolic volume; LVEF: left ventricular ejection fraction; LVESV: left ventricular end-systolic volume; MRA = mineralocorticoid receptor antagonist; NYHA = New York Heart Association; PCI= percutaneous coronary intervention; PVCs = premature ventricular contractions; RR = Riva Rocci; SD = standard deviation. | | | | |
